# Prevention of Pemetrexed-Induced Rash Using Low-Dose Corticosteroids: A Phase II Study

**DOI:** 10.1093/oncolo/oyab077

**Published:** 2022-03-24

**Authors:** Takumi Sakurada, Hiroshi Nokihara, Tadashi Koga, Yoshito Zamami, Mitsuhiro Goda, Kenta Yagi, Hirofumi Hamano, Fuka Aizawa, Hirokazu Ogino, Seidai Sato, Yasushi Kirino, Hisatsugu Goto, Yasuhiko Nishioka, Keisuke Ishizawa

**Affiliations:** Department of Pharmacy, Tokushima University Hospital, Tokushima, Japan; Department of Respiratory Medicine and Rheumatology, Tokushima University Graduate School of Biomedical Sciences, Tokushima, Japan; Clinical Research Professionals, Clinical Study Support, Inc., Nagoya, Japan; Department of Pharmacy, Okayama University Hospital, Okayama, Japan; Department of Pharmacy, Tokushima University Hospital, Tokushima, Japan; Department of Clinical Pharmacology and Therapeutics, Tokushima University Graduate School of Biomedical Sciences, Tokushima, Japan; Clinical Trial Center for Developmental Therapeutics, Tokushima University Hospital, Tokushima, Japan; Clinical Trial Center for Developmental Therapeutics, Tokushima University Hospital, Tokushima, Japan; Department of Pharmacy, Tokushima University Hospital, Tokushima, Japan; Department of Respiratory Medicine and Rheumatology, Tokushima University Graduate School of Biomedical Sciences, Tokushima, Japan; Department of Respiratory Medicine and Rheumatology, Tokushima University Graduate School of Biomedical Sciences, Tokushima, Japan; Department of Pharmacy, Tokushima University Hospital, Tokushima, Japan; Department of Respiratory Medicine and Rheumatology, Tokushima University Graduate School of Biomedical Sciences, Tokushima, Japan; Department of Respiratory Medicine and Rheumatology, Tokushima University Graduate School of Biomedical Sciences, Tokushima, Japan; Department of Pharmacy, Tokushima University Hospital, Tokushima, Japan; Department of Clinical Pharmacology and Therapeutics, Tokushima University Graduate School of Biomedical Sciences, Tokushima, Japan

**Keywords:** Rash prevention, low-dose dexamethasone, pemetrexed, non-squamous non–small cell lung cancer, malignant pleural mesothelioma

## Abstract

**Background:**

Rash eruptions are a common side-effect of pemetrexed, for which the administration of 8 mg/day of dexamethasone for 3 days from the day preceding pemetrexed administration is recommended. This study aimed to prospectively assess the effectiveness of prophylactic administration of low-dose dexamethasone for pemetrexed-induced rashes.

**Methods:**

This single-arm, phase II study recruited patients with non-squamous non–small cell lung cancer and malignant pleural mesothelioma scheduled to receive chemotherapy including pemetrexed. Patients received 2 mg of dexamethasone daily from days 2 to 6 after chemotherapy with pemetrexed. The primary endpoint was the 3-week incidence of rash eruptions.

**Results:**

Twenty-five patients were enrolled between September 2017 and May 2019. The incidence of rash after 3 weeks was 16.7%. Rashes erupted mainly on the upper half of the body, such as the chest and neck, and were of grades 1 and 2 in 2 patients each. No rashes of grade 3 or higher were observed, and there were no adverse events associated with additional corticosteroids.

**Conclusion:**

Prophylactic administration of low-dose dexamethasone for 5 days from the day after pemetrexed administration resulted in a milder incidence and severity of rash. These findings may provide a standard preventative strategy for pemetrexed-induced rashes. (Trial identifier: UMIN000025666).

## Teaser

Rash eruptions are a common side effect of pemetrexed. This article assesses the effectiveness of prophylactic administration of low-dose dexamethasone for pemetrexed-induced rashes.

**Lessons Learned**

- This trial focused on pemetrexed-induced rash occurrence with low-dose corticosteroids.
- The incidence of rash after pemetrexed administration was 16.7%.
- The incidence and severity of rash were mild with low-dose corticosteroids.
- These findings may provide standard preventative measures for pemetrexed-induced rash.

## Discussion

**Figure 1.**
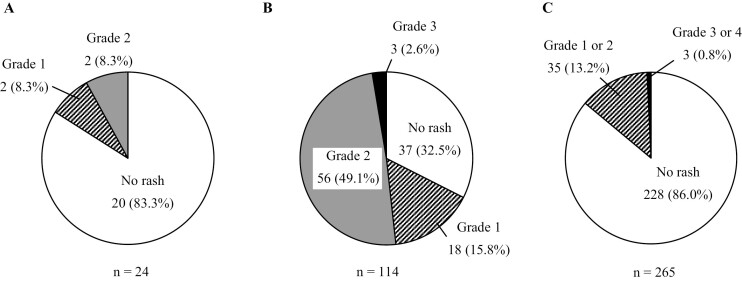
Proportions of patients with rash in the present and previous studies. (**A**) The present study, (**B**) a previous phase II clinical study of pemetrexed alone without systemic corticosteroids for managing non–small cell lung cancer (NSCLC), and (**C**) a previous phase III clinical study of pemetrexed alone with oral dexamethasone 4 mg twice daily the day before, the day of, and the day after pemetrexed administration for managing NSCLC. In (**B**) and (**C**), the rash grade was evaluated based on the Common Terminology Criteria for Adverse Events version 3.0.

Pemetrexed is a key agent for non-small cell lung cancer (NSCLC) and malignant pleural mesothelioma (MPM). A common side effect of pemetrexed is rash eruptions, for which 8 mg/day of dexamethasone is recommended for 3 days from the day preceding pemetrexed administration. Our previous study showed that low-dose corticosteroids could prevent pemetrexed-induced rash. This study aimed to assess the supplementary administration of low-dose dexamethasone after pemetrexed administration. We administered 2 mg of dexamethasone per day for 5 days beginning on the second day after pemetrexed administration and investigated the incidence of rash. Twenty-five patients who received pemetrexed for the first time were enrolled between September 2017 and May 2019. The primary endpoint of rash within 3 weeks after pemetrexed administration occurred in 4/24 (16.7%) patients (95% CI: 4.7-37.4) (Fig. 1). Rashes erupted mainly on the upper half of the body, such as the chest and neck, and were of grades 1 and 2 in 2 patients each. Considering the rash incidence rates of 14.0% (95% CI: 10.0-18.7) and 67.5% (95% CI: 58.1-76.0) in historical control patients treated with and without corticosteroids, respectively, the incidence of rash in this study was not high (Fig. 1). We examined the relationship between rash development and patient background and determined that the number of previous chemotherapy cycles was a significant factor, with an odds ratio of 3.30 (95% CI: 1.15-13.35; Table 1). We believe that our study makes a significant contribution to the literature because the efficacy of other administration protocols has not been examined, as a daily dose of 8 mg of dexamethasone is high and corticosteroids can cause clinical problems, such as hyperglycemia and mental disorders, among others. Therefore, we deemed it necessary to demonstrate the optimal dosage of corticosteroids for the prevention of eruptions induced by pemetrexed. This study revealed that low-dose corticosteroids (2 mg/day) suppressed rash development in patients with non-squamous NSCLC or MPM with an efficacy comparable with that of high-dose corticosteroids. These findings may provide a standard preventative strategy for pemetrexed-induced rash.

## Trial Information

**Table:** 

Disease	Lung cancer—NSCLC; mesothelioma
Stage of disease/treatment	Metastatic/advanced
Prior therapy	No designated number of regimens
Type of study	Phase II, single arm
Primary endpoint	3-week incidence of rash eruptions
Investigator’s Analysis	Active and should be pursued further

### Additional Details of Endpoints or Study Design

#### Patients

Patients with histologically or cytologically proven non-squamous NSCLC or MPM who had never received pemetrexed and were scheduled to undergo chemotherapy with pemetrexed were considered eligible. The eligibility criteria included age ≥18 years; Eastern Cooperative Oncology Group performance status of 0-2; treatment with folic acid and vitamin B_12_ ≥1 week before the day of pemetrexed administration; and adequate hematopoietic (neutrophil count ≥1500/mm^3^, hemoglobin concentration ≥9.0 g/dL, and platelet count ≥100 000/mm^3^), hepatic (total bilirubin concentration ≤1.5 times the upper limit of normal of the institutional reference range [ULN], aspartate aminotransferase, and alanine aminotransferase concentrations ≤3 times the ULN), and renal (creatinine clearance ≥45 mL/minute) function within 14 days prior to enrolment.

We excluded patients who received pemetrexed concomitantly with cisplatin because they required >8 mg of dexamethasone per day for antiemetic prophylaxis for 4-5 days. Patients with active infection, those with severe diabetes mellitus for whom corticosteroid administration was contraindicated, those who required systemic antihistamines and corticosteroids, and those with positive test results for the hepatitis B antigen and anti-hepatitis C antibody were excluded.

The study protocol was reviewed and approved by the Ethics Committee of Tokushima University Hospital in Tokushima, Japan. The work has been carried out in accordance with The Code of Ethics of the World Medical Association (Declaration of Helsinki). All participants provided written informed consent prior to enrolment.

#### Study Design

The present single-arm study was conducted at Tokushima University Hospital. To prevent nausea, on the day of pemetrexed administration (day 1), we intravenously administered 9.9 mg of dexamethasone combined with carboplatin or 6.6 mg of dexamethasone without carboplatin. From days 2-6, 2 mg of dexamethasone was administered orally to prevent rash.

The primary endpoint was the incidence of rash after pemetrexed administration compared with historical controls. The observation period was 21 days after pemetrexed administration, which covered one cycle of the targeted chemotherapy regimen used in this study.

Complete blood counts and biochemical profiles were assessed, and physical assessments were performed within 7 days of study initiation and 21 days after pemetrexed administration. The rash was evaluated once before pemetrexed administration, ≥4 times during days 1-14, as needed on days 14-21, and once after the first cycle. Adverse events were monitored for 21 days after pemetrexed administration. Incident adverse events that did not improve during this period were followed up until improvement.

### Statistical Analyses

This study aimed to accurately estimate the rate of rash development after pemetrexed administration in the context of combined low-dose corticosteroid administration. We assumed a rash occurrence rate of 0.1 (10%) and a half-width of the two-sided 95% Wilson score confidence interval of 0.15 (15%). With 23 cases, the conditional probability was calculated as 0.807. Considering ineligible cases, we decided that 25 patients were required for this study. Rash incidence was summarized in terms of counts, percentages, and 95% confidence intervals at 3 weeks. To assess the relationship between rash incidence and each patient characteristic and adverse effect, an appropriate statistical test (eg, Fisher’s exact test, logistic regression analysis, or Spearman’s rank correlation coefficient) was performed. We used data from two clinical trials—one phase II trial of pemetrexed without corticosteroids and one phase III trial of pemetrexed with corticosteroids (oral dexamethasone 4 mg twice daily the day before, the day of, and the day after pemetrexed)—as historical controls for comparison. The incidence of rash in lung cancer patients treated with pemetrexed is reportedly 67.5% without corticosteroids and 14.0% with corticosteroids. All statistical analyses were performed using JMP version 14.0 (SAS Institute Inc., Cary, NC). A *P*-value of <.05 was considered statistically significant.

## Drug Information

**Table:** 

Generic/working name	Pemetrexed
Trade name	Alimta
Company name	Eli Lilly & Co.
Drug type	Antimetabolite
Dose	500 mg/m^2^
Route	i.v.
Schedule of administration	Pemetrexed was given on day 1

Generic/working name	Dexamethasone
Trade name	Decadron
Company name	Nichi-Iko Pharmaceutical Co., Ltd.
Drug type	Steroid
Dose	2 mg/day
Route	Oral (p.o.)
Schedule of administration	Dexamethasone was administered from days 2 to 6 after chemotherapy with pemetrexed

## Patient Characteristics

**Table:** 

Number of patients, male	11
Number of patients, female	13
Stage	IIIA/IIIB—5 IVA/B—16 Recurrence—3
Age	Median (range): 69.5 (48-83) years
Number of prior systemic therapies	Median (range): 3 (0-3)
Performance status: ECOG	0—5 1—16 2—3 3—0 Unknown—0
Other	Smoking status: never, 8; Ex/current, 16 Line of treatment: first line, 11; second line, 7; greater than or equal to third line, 6Regimen: PEM alone, 9; PEM+ CBDCA, 9; PEM+Bevacizumab, 4; PEM+CBDCA+Bevacizumab, 2 Twenty-five patients who received pemetrexed for the first time were enrolled between September 2017 and May 2019. One enrolled patient was excluded from all analyses due to progression to PS 3 caused by increased pleural effusion during pemetrexed administration. Eleven (45.8%) patients were >75 years of age. Many patients had a good PS, with 87.5% of patients having a PS of 0-1. Pemetrexed was frequently administered as first-line therapy (11/24 patients, 45.8%), with carboplatin (6/11 patients, 54.5%), bevacizumab (2/11 patients, 18.2%), or as a single agent (3/11 patients, 27.3%). Seven (29.2%) patients had previously experienced rash after receiving epidermal growth factor receptor tyrosine kinase inhibitors.
Cancer types or histologic subtypes	Non–small cell lung cancer, 21; malignant pleural mesothelioma, 3

## Primary Assessment Method

**Table:** 

Title	The incidence of rash after pemetrexed administration
Number of patients screened	25
Number of patients enrolled	25
Number of patients evaluable for toxicity	24
Number of patients evaluated for efficacy	24
Evaluation Method	National Cancer Institute Common Terminology Criteria for Adverse Events version 4.0

### Outcome Notes

Rash within 3 weeks after pemetrexed administration occurred in 4/24 (16.7%) patients (95% CI: 4.7-37.4). Of these, 8.3% had rashes classified as grade 1 or 2 (Fig. 1). The rash incidence in this study was low compared with the rate of 67.5% (95% CI: 58.1-76.0) reported for patients treated without corticosteroids and did not increase compared to the rate of 14.0% (95% CI: 10.0-18.7) reported in patients treated with corticosteroids. We also examined the relationship between rash development and patient background and determined that the number of previous chemotherapy lines was a significant factor, with an odds ratio of 3.0 (95% CI: 1.14-12.85; Table 1).

**Table 1.** Relationship between clinical variables and pemetrexed-induced rash.

Variable	Rash		OR	(95% CI)
	None (*n* =20 )	Incidence (*n* = 4)		
Age, years, median (range)	72.5 (48-83)	67.5 (64-80)	1.00	(0.91-1.12)
Sex (M/F), *n*	9/11	2/2	1.23	(0.13-11.93)
ECOG PS (0/1/2), *n*	5/12/3	0/4/0	1.37	(0.21-10.21)
CrCl (mL/minute), median (range)	62 (48-97.8)	71.6 (53.4-112.3)	1.04	(0.98-1.11)
History of EGFR-TKI usage, *n* (%)	5 (25.0%)	2 (50.0%)	3.00	(0.3-31.15)
Previous lines of chemotherapy (0/1/2/3), *n*	10/7/2/1	1/0/1/2	3.21	(1.14-12.85)
Carboplatin combination, *n* (%)	10 (50.0%)	1 (25.0%)	0.34	(0.02-3.13)
Bevacizumab combination, *n* (%)	5 (25.0%)	1 (25.0%)	1.00	(0.05-10.14)
NSAID combination, *n* (%)	4 (20.0%)	1 (25.0%)	1.34	(0.06-14.24)

Abbreviations: CI, confidence interval; CrCl, creatinine clearance; ECOG PS, Eastern Cooperative Oncology Group performance status; EGFR-TKI, epidermal growth factor receptor tyrosine kinase inhibitor; F, female; M, male; NSAID, non-steroidal anti-inflammatory drug; OR, odds ratio.

## Adverse Events, Cycle 1

**Table:** 

Name	*NC/NA	1	2	3	4	5	All grades
Neutrophil count decreased	58%	0%	25%	13%	4%	0%	42%
Anemia	29%	50%	17%	4%	0%	0%	71%
Platelet count decreased	50%	25%	4%	17%	4%	0%	50 %
Febrile neutropenia	96%	0%	0%	4%	0%	0%	4%
Alanine aminotransferase increased	58%	38%	0%	4%	0%	0%	42%
Aspartate aminotransferase increased	58%	38%	4%	0%	0%	0%	42%
Vomiting	100%	0%	0%	0%	0%	0%	0 %
Nausea	96%	4%	0%	0%	0%	0%	4%
Anorexia	83%	13%	4%	0%	0%	0%	17%
Fatigue (asthenia, lethargy, malaise)	83%	17%	0%	0%	0%	0%	17%
Mucositis oral	92%	8%	0%	0%	0%	0%	8%
Creatinine increased	92%	8%	0%	0%	0%	0%	8%

There were no serious nonhematological adverse events (Table 2), see also Table 3. Although the carboplatin combination regimen was associated with a moderate risk of emesis, palonosetron hydrochloride (0.75 mg) and dexamethasone (9.9 mg) were administered on day 1 without fosaprepitant dimeglumine in the present study. No vomiting was observed, and grade 2 nausea or anorexia was observed in one patient. As expected, serious hematological toxicity occurred at a greater rate in the carboplatin combination group, and adverse effects of grade ≥3 were observed in 2/13 (15.4%) patients in the carboplatin noncombination group and 5/11 (45.5%) patients in the carboplatin combination group. Compared with the results of previous clinical trials, myelosuppression occurred at a similar rate in the carboplatin noncombination group but was more frequently observed in the carboplatin combination group.

**Table 2.** Characteristics of patients with rash.

Patient	Grade of rash	Chemotherapy	Development of rash	Location of rash	Treatment	Outcome
1	2	PEM	Day 3	Face, neck, both forearms	None	Cure
2	1	PEM + CBDCA	Day 6	Chest	Topical steroids	Cure
3	1	PEM + bevacizumab	Day 16	Neck, chest	Moisturizing agent	Cure
4	2	PEM	Day 8	Chest, abdomen	None	Cure

Abbreviations: CBDCA, carboplatin; PEM, pemetrexed.

**Table 3.** Adverse events occurring within 3 weeks after pemetrexed administration.

Event	PEM ± bevacizumab (*n* =13)						PEM + CBDCA ± bevacizumab (*n* =11)					
CTCAE grade	1	2	3	4	Grade 3/4 (%)		1	2	3	4	Grade 3/4 (%)	
Hematological												
Neutrophil count decreased	0	1	2	0	2	(15.4)	0	5	1	1	2	(16.7)
Anemia	6	2	0	0	0	(0.0)	6	2	1	0	1	(8.3)
Platelet count decreased	3	0	1	0	1	(7.7)	3	1	3	1	4	(33.3)
Febrile neutropenia	0	0	0	0	0	(0.0)	0	0	1	0	1	(8.3)
Nonhematological												
Elevated AST/ALT level	6	0	0	0	0	(0.0)	4	0	1	0	1	(8.3)
Vomiting	0	0	0	0	0	(0.0)	0	0	0	0	0	(0.0)
Nausea/anorexia	1	0	0	0	0	(0.0)	3	1	0	0	0	(0.0)
Fatigue	3	0	0	0	—	(0.0)	1	0	0	0	0	(0.0)
Oral mucositis	1	0	0	0	0	(0.0)	1	0	0	0	0	(0.0)
Elevated creatinine level	1	0	0	0	0	(0.0)	1	0	0	0	0	(0.0)
Transient fever	0	0	0	0	0	(0.0)	0	0	0	0	0	(0.0)

Abbreviations: ALT, alanine aminotransferase; AST, aspartate aminotransferase; CBDCA, carboplatin; CTCAE, Common Terminology Criteria for Adverse Events; PEM, pemetrexed.

## Assessment, Analysis, and Discussion

**Table:** 

Completion	study completed
Investigator’s Assessment	Active and should be pursued further

Pemetrexed exhibits high antitumor activity in non-squamous NSCLC and MPM and is a key drug in the treatment of these neoplasms.^1-5^ Although skin rash is among the most common adverse events resulting from pemetrexed administration, strategies for preventing rash remain to be clarified. According to previous clinical studies,^1,6^ pemetrexed-induced rash can be prevented by administering 4 mg of dexamethasone per o.s. twice daily for 3 days (a total of 24 mg) from the day preceding pemetrexed administration. However, a daily dose of 8 mg of dexamethasone is high, as corticosteroids can cause clinical problems such as hyperglycemia, arrhythmia, hypertension, mental disorders, and edema. Our previous retrospective study suggested that the effects of corticosteroids on pemetrexed-induced rash are not dose dependent and that low doses of corticosteroids may yield sufficient effects.^7^ We conducted the present phase II study to evaluate the effectiveness of low-dose corticosteroids in preventing pemetrexed-induced rash in patients with NSCLC and MPM. We administered 2 mg of dexamethasone per day for 5 days beginning on the second day after pemetrexed administration and investigated the incidence of rash. In our present study, four of 24 (16.7%) patients developed pemetrexed-induced rash, while 77/114 (67.5%) patients developed rash in a phase II study^5^ of pemetrexed treatment alone for NSCLC without corticosteroid administration (Fig. 1). Grade 2 rash also occurred frequently in the phase II study^5^—49.1% compared with 8.3% in this study. Additionally, in a phase III study^2^ of pemetrexed for managing NSCLC, in which high doses of corticosteroids were administered to all patients as a prophylactic measure against rash, the incidence of rash was 14.0% (Fig. 1). Thus, there was no significant increase in the incidence of rash in this study compared with those in previous studies, even when high doses of corticosteroids were concomitantly administered.

In clinical studies of pemetrexed without corticosteroids,^1,5,6,8^ the reported incidence of rash was 67.5%-93%. In clinical studies of patients treated with high doses of corticosteroids,^1,2,6^ the incidence of rash was 14.0-56.0%. Based on these findings, the incidence of rash in our study did not increase even when the dose of corticosteroids was reduced, implying that 2 mg/day of dexamethasone for 5 days is likely to be effective in preventing rash after pemetrexed administration. The rashes that developed in this study were mild, and most cases resolved spontaneously or disappeared immediately among patients who used topical steroids (Table 2). None of the cases affected the continuation of chemotherapy with pemetrexed.

Recently, immune checkpoint inhibitors such as programmed death 1/programmed death ligand 1 (PD-1/PD-L1) inhibitors have been used in the treatment of lung cancer.^9-12^ For patients with advanced non-squamous NSCLC, treatment with PD-1/PD-L1 inhibitors plus platinum and pemetrexed is widely used as a standard of therapy.^11,12^ Arbour et al investigated the effect of corticosteroids used at the initiation of treatment on the antitumor effect of PD-1/PD-L1 blockade in patients with non–small cell lung cancer.^13^ They reported that baseline treatment with corticosteroids was associated with lower overall response rates and progression-free survival and recommended that corticosteroids should be used with caution when using PD-1/PD-L1 blockade.^13^ Considering these findings, the amount of corticosteroids used to prevent rash during treatment with PD-1/PD-L1 inhibitors plus platinum and pemetrexed should remain as little as possible.

In this study, we observed that the incidence of rash after pemetrexed administration was associated with number of previous lines of chemotherapy (Table 1). Studies have indicated that cancer progression may induce an inflammatory response through several mechanisms induced by inflammatory cytokines and reactive oxygen species.^14,15^ Similarly, rash may be caused by various factors such as an inflammatory response. Thus, it is possible that patients undergoing late-line chemotherapy exhibit increased cancer momentum due to ineffective pretreatment, making them more susceptible to rash. However, we did not investigate factors associated with rash development in this study. A study with a large sample size is required to elucidate these factors and determine whether the number of previous lines of chemotherapy is related with them. Following previous lines of chemotherapy, the odds ratio for a history of epidermal growth factor receptor tyrosine kinase inhibitor (EGFR-TKI) usage was as high as 3.0, although not significant (Table 1). It would be interesting to further investigate whether a history of EGFR-TKI usage affects the development of pemetrexed-induced rash.

We recognize that our study has several limitations. This single-arm study had no control group and used indirect historical controls instead. Two major clinical studies of pemetrexed in Japan,^4,5^ which we used as historical controls, investigated the incidence of rash, and assessed its grade according to the Common Terminology Criteria for Adverse Events. Single-arm studies have several shortcomings, such as limitations on the generalizability of their findings to other populations and may not be appropriate for comparison with other studies.^16^ There were also differences in patient characteristics, such as concomitant medications and prior history of chemotherapy, which may have influenced the development of rash after pemetrexed administration. Furthermore, the number of cases was small, and the effects of factors other than pemetrexed on the occurrence of rash may have influenced the results. Additionally, factors that may suppress the development of rash, such as the use of systemic corticosteroids other than those used in the present study and antihistamines, were excluded. We consider that a potential bias may have arisen regarding the causes of rash.

To the best of our knowledge, there are no clinical trials that have prospectively investigated the development of frequent rashes after administration of pemetrexed as the primary endpoint. We believe that these findings provide standard data on the development of pemetrexed-induced rash, and that the method of corticosteroid administration used in this study is superior and more versatile than the currently recommended method, which involves the administration of high-dose corticosteroids for 3 days following pemetrexed administration.

## Data Availability

The data underlying this article will be shared on reasonable request to the corresponding author.
